# Nonpoint Pollution Source-Sink Landscape Pattern Change Analysis in a Coastal River Basin in Southeast China

**DOI:** 10.3390/ijerph15102115

**Published:** 2018-09-26

**Authors:** Xin Zhang, Qiong Zheng, Lin Zhou, Jiawei Wei

**Affiliations:** 1 State Key Laboratory of Remote Sensing Science, Institute of Remote Sensing and Digital Earth, Chinese Academy of Sciences, Beijing 100101, China; 2 College of Geoscience and Surveying Engineering, China University of Mining and Technology (Beijing), Beijing 100083, China; zhengqiong90@163.com; 3 College of Remote Sensing Information Engineering, Wuhan University, Wuhan 430079, China; 2015302590149@whu.edu.cn; 4 College of Geomatics Science and Technology, Nanjing Tech University, Nanjing 211816, China; authurwei@foxmail.com

**Keywords:** landscape pattern, change, source-sink, random forest, landscape metrics, transition matrix

## Abstract

Analyzing the spatiotemporal characteristics of source-sink landscape pattern change in river basins is crucial for managing and controlling nonpoint source pollution. This study investigated the landscape pattern changes in Jiulong River basin from 1990 to 2015. A random forest classifier combined with texture and spectral information was applied to interpret the multi-temporal Landsat images. Landscape metrics were calculated to quantify the landscape at the patch level. Transition matrixes were derived for analyzing the conversion among different landscape types. It is notable that the largest values of the number of patches and patch density of residential land appeared in 2005, indicating the highest degree of fragmentation over this time period. The percentage of landscape for forestland was always higher than 71%, and the percentage of residential land increased from 7.42% to 14.55% during the last three decades, while unused land decreased from 5.3% to 2.8%. The downward trend of DO and the upward trend of NH_3_-N and TP indicate the deterioration of water quality during 2005–2015. The quantitative monitoring data of water quality indicators in Hua’an and Xiamen sites in Jiulong River basin are shown. The percentage of landscape of cultivated land increased during 2005–2010, which was consistent with the change tendency of NH_3_-N. Transition matrixes showed that the main changes occurred when forestland and unused land were transformed to residential land and cultivated land over the last three decades. Analysis results demonstrated a higher extent of landscape fragmentation and an unsustainable transition among source-sink landscapes.

## 1. Introduction

Strong links exist between landscape patterns and ecological function and process [1]. Landscape structure and composition evolve continuously in space and time [2]. Analysis of landscape pattern change is a key issue in landscape ecology. Non-point source pollution originates from diffuse areas where some solvable or solid pollutants are produced, and it is more severe compared to point source pollution [3]. Non-point source pollution has been the main cause of water quality deterioration with regards to urbanization and industrialization [4]. On the basis of source-sink theory, some landscape types contribute significantly to the pollution, which can be categorized as source landscapes, while some landscape types can reduce the pollutants and are defined as sink landscapes [5]. Chen found that the spatial distribution of source-sink landscapes is closely related with the generation of non-point source pollution [6]. Liu simulated the non-point source pollution load in urban planning, historic trends and ecological protection land use scenarios based on the Conversion of Land Use and its Effect at Small regional extent (CLUE-S) and Soil and Water Assessment Tool (SWAT) models and the results reflected that land use had an impact on non-point source pollution [7]. Choi pointed out that land use changes influenced non-point source pollutant loads significantly, since land use changes modified the sources of pollutants and the way they were carried to streams [8]. Previous studies have demonstrated the correlation between land use change and non-point source pollution load [9]. Jiang analyzed the correlation between the source and sink structure and the non-point source pollution by calculating the location-weighted Landscape Contrast Index (LCI) and the Modified Grid source-sink Landscape Contrast Index (mGLCI), and it was shown that an appropriate increase in the sink landscape can inhibit the risk of non-point source pollution affecting the water quality [10]. Qualitative and quantitative analysis of the characteristics of source-sink landscape pattern evolution can provide strong support for the control of nonpoint source pollution and can be employed for future land-use allocation [7]. Watershed land use affects water quality through non-point source (NPS) pollutants, which are major contributors of contaminants to the catchment-coast continuum [11]. Therefore, exploring the linkage between land use and surface water quality, particularly in coastal watersheds, is critical for developing watershed management practices and controlling land-based pollution in coastal bays [12,13].

In this paper, we use multi-temporal remote sensing images as fundamental data sets. The key task of this study is the classification of multi-temporal remotely sensed images. Common classification approaches are supervised classifiers and unsupervised classifiers. Supervised approaches need sufficient training samples, and the signatures generated from the reference data are then used to train the classifiers to classify the spectral data into a thematic map, whereas no prior definitions of the classes are used in the unsupervised classifiers. Unsupervised classification methods are clustering-based algorithms [11,12,13]. Unsupervised classifiers and supervised classifiers have been commonly applied for landscape classification in previous studies [14], but their accuracy is not satisfactory because both of them are based on the spectral information for classification. Zhou used a supervised classifier in the study of water quality response to land use change from 1986 to 2010 and the overall accuracy were 81.64% in 1986, 88.67% in 1996, 82.33% in 2002, 83.20% in 2007 and 83.73% in 2010 respectively [15]. Textural image information reflects information about the spatial structure of the objects and their relationship with the surrounding environment. Higher classification accuracy can be obtained through the combination of spectral information with texture features [16]. In the last decade, machine learning algorithms (e.g., neural network ensembles, random forests, bagging and boosting) have emerged as more accurate and efficient alternatives for mapping landscape patterns [17]. A random forest (RF) classifier combined texture with spectral information is applied for landscape classification in this paper. 

Two kinds of quantitative methods are used to study the features of landscape change. One method is to analyze landscape spatial change based on four landscape metrics including number of patches (NP), patch density (PD), mean patch size (MPS) and percentage of landscape (PLAND) [18]. Although some researchers doubt the role of landscape metrics in reflecting the relationship between pattern-processes and while these metrics have limitations, landscape metrics are still important for landscape pattern analysis [19] and can describe the composition and spatial arrangement of landscape types [20]. The other method is to define the mutual transition regimes of various landscape patch types by establishing transition matrixes [21]. The transition matrix is important information for analyzing the temporal and spatial changes of landscape patterns [22]. 

We selected Jiulong River basin as the experimental area. Landsat images covering this area acquired in 1990, 1995, 2000, 2005, 2010 and 2015 were applied as the primary data sets. The RF classifier combined with textural and spectral information was employed to interpret the multi-temporal satellite images identified above. Four landscape metrics were calculated, and the transition matrixes were obtained with the support of IDRISI. Quantified analysis was conducted based on four metrics and transition matrixes. Finally, characteristics of the source-sink landscape pattern change in the study area were obtained.

## 2. Materials and Methods

### 2.1. Study Area and DATA Acquirsion

#### 2.1.1. Study Area

Located in southeast Fujian, China, Jiulong River basin was selected as the study area. Jiulong River is the second-longest river in Fujian Province and has three main streams: North River, West River and South River [23]. The Jiulong River basin covers an area of 14,745 km^2^ bounded between 116°47′ E to 118°02′ E and 24°13′ N to 25°51′ N, and approximately 87.5% of its total area is located in Longyan and Zhangzhou [24]. This area is in a subtropical marine monsoon climate. The mean annual temperature is approximately 20 °C and July is the warmest month while January is the coldest month. Temperature increases from north to south and from coastal to inland regions. The precipitation in the watershed is abundant, with the number of rainy days per year ranging from 100 to 200. The mean annual rainfall ranges from 1400 mm to 1800 mm [25]. Jiulong River is the drinking, industrial and agricultural water source of more than 5 million inhabitants in cities and counties such as Xiamen, Zhangzhou and Longyan. The river’s water quality is thus of great significance to this area. 

#### 2.1.2. Data Source and Preprocessing

According to the scale of watershed and the objectives of this paper, Landsat images with the spatial resolution of 30 m acquired in 1990, 1995, 2000, 2005, 2010 and 2015 were used for landscape classification. Taking a five-year frequency can be beneficial for analyzing the tendency of landscape pattern change in this area, and the data processing complexity is lower than that of a two year or oneyear frequency. The information about sensors of images is showed in Table 1. One image was used for classification each year. ENVI is a platform which is used for processing remote sensing images. The remotely sensed data were imported to ENVI, and Radiance Calibration, FLAASH and Seamless Mosaic in ENVI were applied for preprocessing. Radiance Calibration was used for radiation calibration, FLAASH was applied to atmospheric calibration, and then Seamless Mosaic was employed to obtain the images of the whole watershed.

### 2.2. Source-Sink Landscape

According to the land use classification standards and the actual environment in Jiulong River basin, the watershed was classified into six landscape classes: Residential land, cultivated land, orchards, forestland, water and unused land. The specific definitions are showed in Table 2.

On the basis of landscape ecology, there are strong links between landscape patterns and ecological processes, such as nonpoint source pollution [1,23]. According to source-sink theory, some landscapes in the watershed may serve as pollutant sources for the stream, some may serve as transformation zones, and others may serve as nutrient detention zones [26]. Based on the contributions of the nonpoint source pollution, six major land classes are divided into source landscapes and sink landscapes. Source landscapes, including cultivated land, residential land and orchards, can increase the pollutants. Sink landscapes, which include water, forestland and unused land, inhibit the transmission of nonpoint source pollutants. 

### 2.3. Random Forest Classifier

A RF classifier involves choosing a set of features randomly and creating a classifier with a bootstrapped sample of the training data. A large number of decision trees are generated in this way, and unweighted voting then is used to assign an unknown pixel to a class [27,28,29]. Key advantages of RF include its high classification accuracy, nonparametric nature and ability to determine variable importance [17].

Spectral features describe the average tonal variations in various bands of the visible and infrared portion of an electromagnetic spectrum. Texture features contain information about the spatial distribution of tonal variations within a band [16]. Textural information can be applied to improve the classification accuracy. In this study, an RF classifier combining textural features and spectral information was proposed for landscape classification of remote sensing images, and the results showed that this ensemble classifier had high accuracy. 

**Figure 1:**
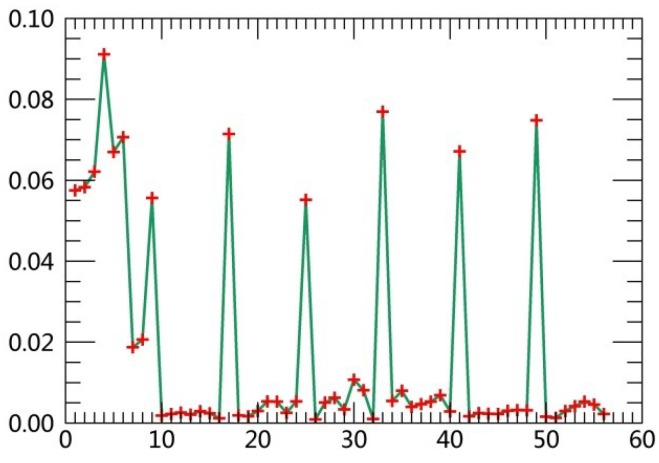
Weight line chart of 54 features in 1990.

**Figure 2:**
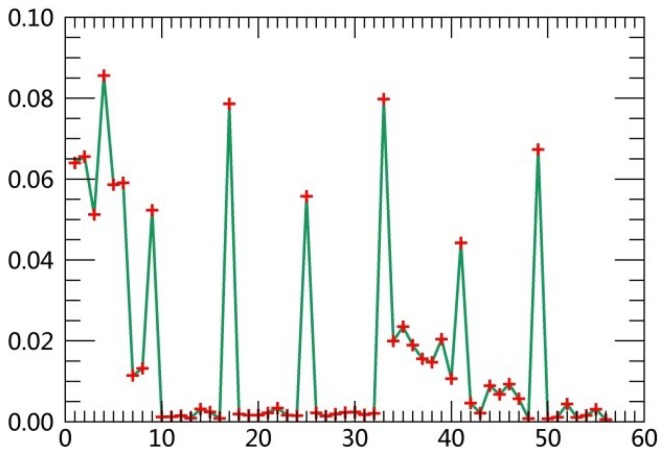
Weight line chart of 54 features in 1995.

**Figure 3:**
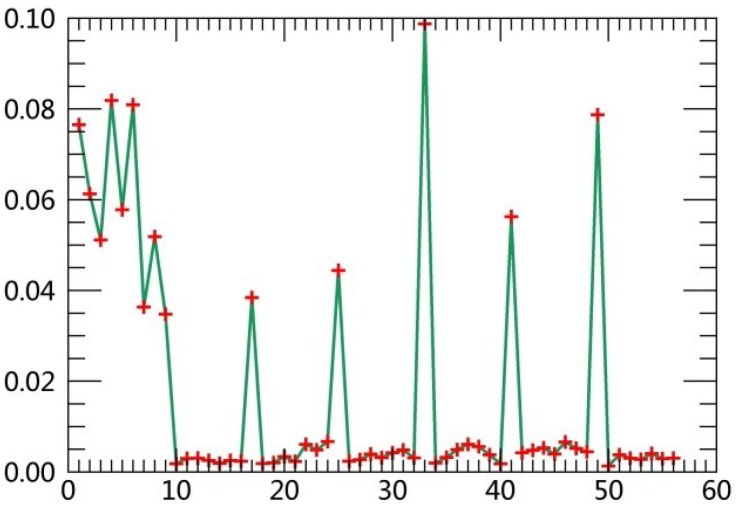
Weight line chart of 54 features in 2000.

**Figure 4:**
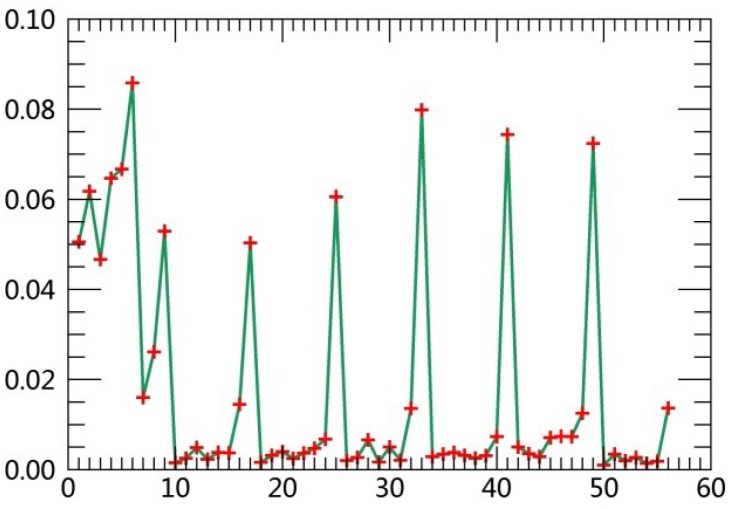
Weight line chart of 54 features in 2005.

**Figure 5:**
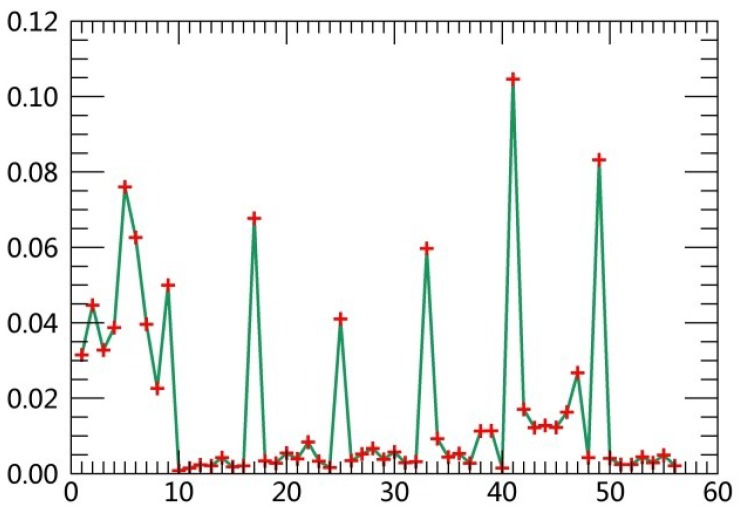
Weight line chart of 54 features in 2010.

**Figure 6:**
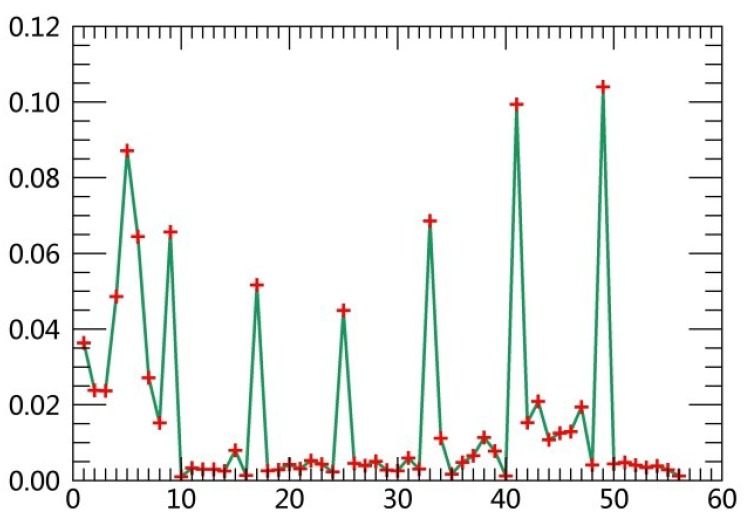
Weight line chart of 54 features in 2015.

The specific steps are described as follows:

(1) Extract class samples based on spectral information. The unsupervised classifier was used for extracting samples of satellite images firstly. The whole watershed was divided into 160 types by ISODATA in ERDAS. Parameters of classification were set as the following: The number of classes was 160; number of maximum iterations was 30; and convergence threshold was 0.95. The results were merged to 6 major classes and the typical patch of each landscape class in final result was extracted for use as regions of interest (ROIs) in the next supervised classification process.
(2) Extract textural information. The co-occurrence measures, provided in the Texture module of ENVI, were employed to extract texture features from remotely sensed images. The scalars characterizing the texture information in the co-occurrence measures include mean, variance, homogeneity, contrast, dissimilarity, entropy, second moment and correlation.
(3) Random forest classification based on textural features and spectral information. Six bands of TM images were used in the unsupervised classifier, and eight textural parameters were calculated based on the Gray-Level Co-occurrence Matrix while extracting texture features. Spectral bands were fused with the logical bands of texture parameters. The Layer Stacking function in ENVI was applied for the fusion of spectral bands and textural features. Using this method, 54 characteristics were obtained. Table 3 displays sequence numbers of 54 characteristics. Weight values of the 54 features were calculated by establishing the decision trees, and then the first 10 features with the largest weights were selected as the characteristics for the image classification. Weight line charts of 54 features of the remote sensing images acquired in 1990, 1995, 2000, 2005, 2010 and 2015 are showed in Figure 1, Figure 2, Figure 3, Figure 4, Figure 5 and Figure 6, and the weight values of the first 10 features in corresponding phases are displayed in Table 4, Table 5, Table 6, Table 7, Table 8 and Table 9. These characteristics were applied as the sample features in the RF classifier. Postprocessing was conducted on the basis of high-resolution images, topographic maps and other information.
(4) Accuracy assessment. The most common methods of accuracy assessment are the confusion matrix and the Kappa coefficient [30]. The confusion matrix is expressed as Equation (1).
  (1) $$M=\left[\begin{array}{cccc} m_{11} & m_{12} & \ldots & m_{1n}\\ m_{21} & m_{22} & \ldots & m_{21}\\ \ldots & \ldots & \ldots & \ldots \\ m_{n1} & m_{n2} & \ldots & m_{\mathrm{nn}} \end{array}\right]\;$$
  where m_ij_ represents the sum of pixels which should be the i type but are divided into the j class, n represents the number of classes, and m_ii_ represents the number of pixels which are classified correctly. The larger the diagonal values are, the higher accuracy the results possess.
  The indexes of accuracy are the Kappa coefficient, the overall accuracy and the user accuracy. The Kappa coefficient and the overall accuracy are employed for classification assessment in this paper. Combined with land planning data of Jiulong River basin and Google Earth, 300 ground checkpoints were randomly selected to calculate the two indexes for accuracy assessments.
  (2) $$Kappa=\frac{P_{0}-P_{e}}{1-P_{e}}\;$$
  Equation (2) gives the value of Kappa, where P_0_ is the percent correct for classification results, and P_e_ is the hypothetical probability of chance agreement.
  (3) $$OA=\frac{n_{c}}{n}\;$$
(5) Equation (3) gives the value of overall accuracy, where n_c_ is the amount of pixels which are classified correctly, and n is number of pixels.

### 2.4. Analysis of Landscape Pattern Change

#### 2.4.1. Change Analysis Based on Landscape Metrics

Landscape pattern analysis is an important topic of landscape ecology [19]. Landscape pattern analysis is used in this paper, which is beneficial for understanding the relationship between landscape patterns and ecological processes along with socio-economic activities. This is of great significance for the rational utilization of landscape resources, ecological landscape construction, land use planning and water quality protection in the basin. 

Four landscape metrics are selected to analyze the landscape change in the Jiulong River basin from 1990 to 2015. The specific descriptions are expressed in Table 10 [31].

A patch is the basic unit of landscape patterns and refers to a relatively homogeneous nonlinear area that is different from the surrounding background. The number of patches (NP) is a simple measure of the extent of fragmentation of the patch type [20]. There is a strong positive correlation between the value of NP and the fragmentation of the landscape [23].

Mean patch size (MPS) refers to the average area of all patches in a landscape type. The extent of fragmentation is positively correlated with MPS. Studies show that changes in MPS can feed back more information about ecological processes and can characterize the subdivision of the landscape, which is key to reflect the heterogeneity of landscapes.

Percentage of landscape (PLAND) is the proportion of a certain landscape type’s area across the whole watershed. Previous research shows that PLAND has more significant correlations with water quality compared with other metrics [23]. 

#### 2.4.2. Transition Matrix of Landscape

The conversion of different landscape types is a crucial issue in the study of landscape ecology. All or part of a landscape type may be transferred to other landscape types under the influence of human activities and natural process, yielding distinct changes in landscape patterns. The Crosstab module in IDRISI was used to calculate the cross-tabulation table, which was output as a transition matrix.

## 3. Results and Analysis

### 3.1. Classification Results

The Kappa coefficient and the overall accuracy of classification results for each year were calculated by the methods listed in Section 2. We displayed the results in Table 11.

The overall accuracy for each year is more than 86%, and the Kappa coefficients are all larger than 0.83. The RF classification has a relatively high accuracy compared with former classifiers. A Supervised Maximum Likelihood classification algorithm was applied in multi-temporal Landsat images of Avellino, and the overall classification accuracy and Kappa indexes were not at a stable level: The lowest accuracy and Kappa reached 82.42% and 0.6863, while the highest were 95.70% and 0.9285, respectively [20]. The unsupervised method was used for landscape classification in Jiulong River basin and the overall Kappa coefficients were 71% for 2002 and 74.53% for 2007 [23]. The overall accuracy of the random forest classifier employed in this study was relatively higher than some normal classification algorithms used in previous research, and the overall accuracy and Kappa indexes for multi-temporal images remained stable.

**Figure 7:**
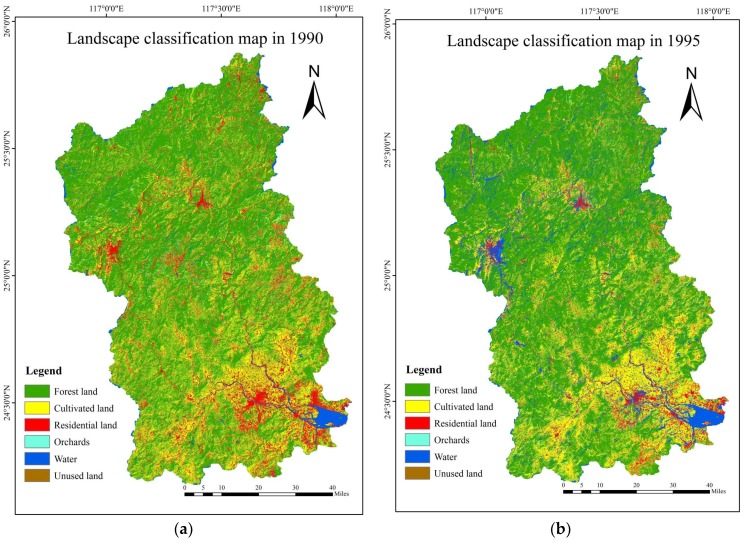

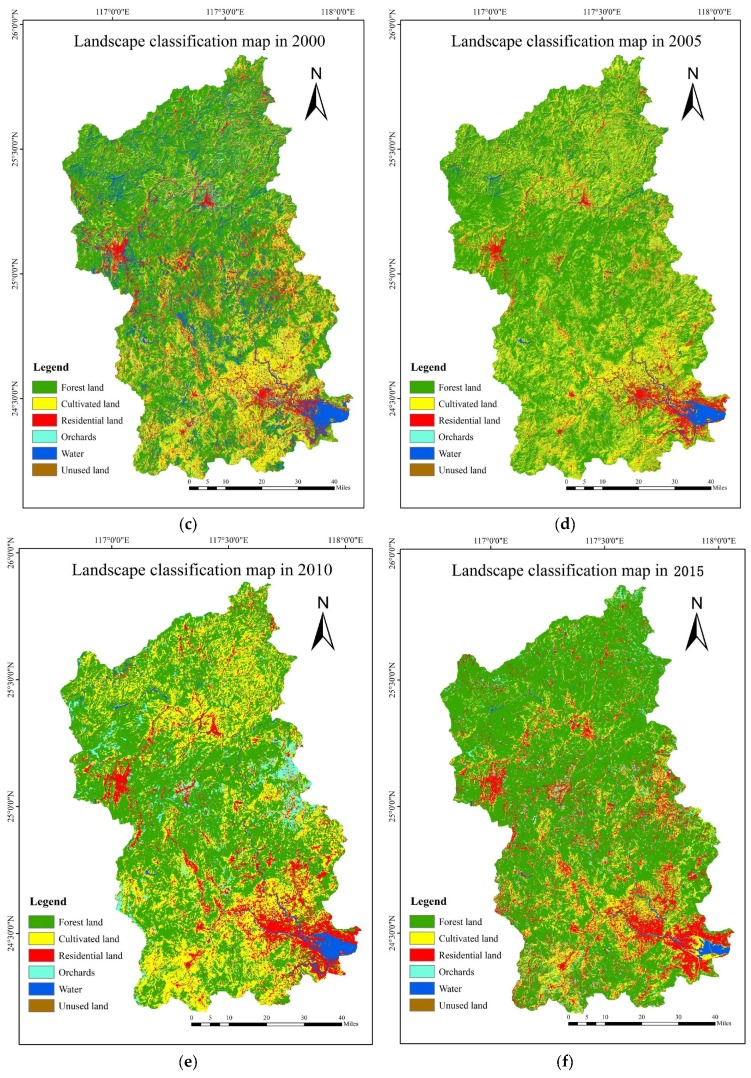
The multi-temporal classification figure of the source-sink landscape pattern in the Jiulong River basin. (**a**) Landscape classification map in 1990. (**b**) Landscape classification map in 1995. (**c**) Landscape classification map in 2000. (**d**) Landscape classification map in 2005. (**e**) Landscape classification map in 2010. (**f**) Landscape classification map in 2015.

Based on the classification results of the remote sensing images, the landscape pattern figures of 1990, 1995, 2000, 2005, 2010 and 2015 (Figure 7) were produced by using ArcGIS 10.1 (Esri, Redlands, CA, USA).

The area of each landscape type in different years was calculated through attribute commands in the ArcGIS platform based on the classification figures. Table 12 represents the area of source-sink landscape types of Jiulong River watershed in 1990, 1995, 2000, 2005, 2010 and 2015. 

**Figure 8:**
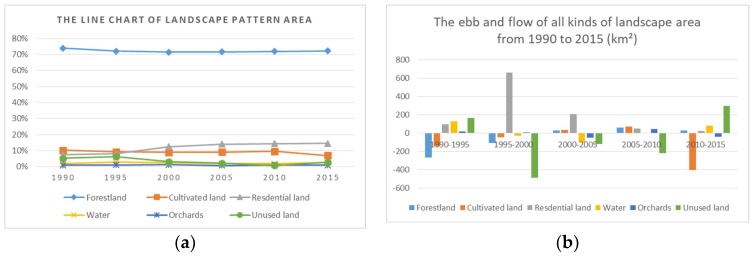
(**a**) The line chart of landscape pattern area percentage. (**b**) The ebb and flow of all landscape type areas from 1990 to 2015.

Table 12 and Figure 8 show the increase of area in residential land and the decrease of area in cultivated land and unused land during 1990–2015. The area of residential land had the largest increase from 1179.27 km^2^ to 1838.68 km^2^ during 1995–2000; in contrast, the area of unused land decreased from 935.55 km^2^ to 450.09 km^2^ in this period. Additionally, the area of residential land increased constantly but with a progressively slower trend. The area of cultivated land changed rapidly during 2010–2015, decreasing from 1416.94 km^2^ to 1016.12 km^2^. Forestland occupied at least 70% of the total area and is the dominant landscape type in Jiulong River basin. The area of forestland began to increase after 2000, which was result of the Grain to Green governmental policy. On the whole, the area of source landscapes increased notably during 1990–2015, while the area of sink landscapes had an overall decreasing trend.

### 3.2. Landscape Metrics Analysis

Four landscape metrics were calculated based on the classified multi-temporal remote sensing images of Jiulong River basin by using ArcGIS. Landscape pattern analysis was performed on the basis of four landscape metrics.

As shown in Table 13 and Table 14, PD had a similar variation tendency compared to NP for each landscape type, which was directly related to the calculation formulas of them. PD was also synchronous with NP. Both PD and NP can reflect the extent of landscape fragmentation. The increase of PD or NP indicated that the landscape was more fragmented than before and vice versa. MPS can represent the subdivision of the landscape as well, but it has the opposite tendency to PD and NP. 

The values of PD and NP of residential land were largest in 2005 and smallest in 1995, which indicated that the highest degree of fragmentation in residential land appeared in 2005 and the lightest fragmentation appeared in 1995. According to Table 6, the change tendencies of cultivated land had the same features as residential land. The PD and NP values of unused land decreased rapidly from 1995 to 2010, mainly because of the rapid development of urbanization and industrialization in the Jiulong River drainage area. 

We found that the MPS of forestland decreased during 1990–2000 in Table 15, which indicated a higher degree of fragmentation, and then MPS values increased from 2000 to 2015, representing reduced subdivision. In particular, the MPS values of residential land demonstrated a constantly increasing tendency. 

According to Table 16, the PLAND of residential land increased constantly, and the largest increase (from 8.10% to 12.62%) appeared after 1995, while the PLAND of unused land decreased rapidly (from 5.30% to 2.80%) during the study periods, which corresponded with the characteristics of urbanization processes in the study area. The PLAND of forestland was the largest and remained stable during the last three decades. The PLAND of cultivated land was distinctly reduced (from 9.72% to 6.98%) during 2010–2015, but in contrast, the PLAND of unused land had an increase from 0.77% to 2.80% during the same period.

**Figure 9:**
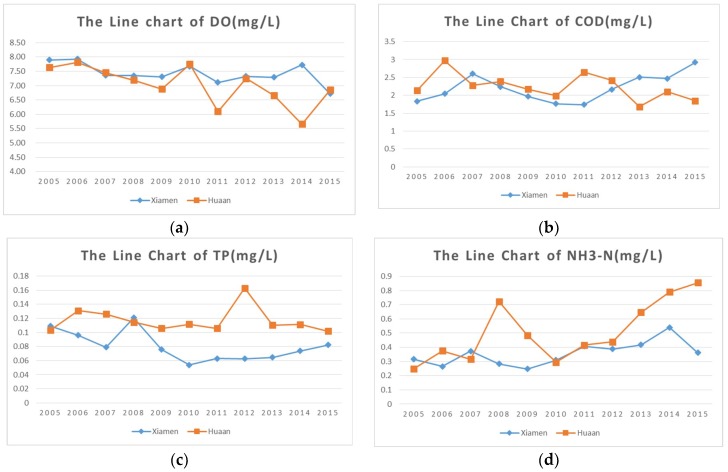
(**a**) The line chart of DO monitoring data in Xiamen and Hua’an from 2005 to 2015. (**b**) The line chart of COD_Mn_ monitoring data in Xiamen and Hua’an from 2005 to 2015. (**c**) The line chart of TP monitoring data in Xiamen and Hua’an from 2005 to 2015. (**d**) The line chart of NH_3_-N monitoring data in Xiamen and Hua’an from 2005 to 2015.

The monitoring data of water quality indicators in Hua’an and Xiamen sites in Jiulong River basin, obtained from the weekly report of water quality on the official website of the Fujian Provincial Department of Environment Protection, were showed in Table 17 and the monitoring indices of water quality include pH, dissolved oxygen (DO), chemical oxygen demand (COD_Mn_), total phosphorus (TP) and ammonia nitrogen amount (NH_3_-N). The changes of four indicators from 2005 to 2015 were displayed in the form of line charts in Figure 9. Zhang established the multiple linear regression models of water quality indicators and landscape metrics and results demonstrated the salient correlation between water quality indicators (including TP, COD_Mn_ and NH_3_-N) and landscape metrics [32]. The downward trend of DO and the upward trend of NH_3_-N and TP indicate the deterioration of water quality during 2005–2015. PLAND of cultivated land increased slightly during 2005–2010, which was consistent with the change tendency of NH_3_-N. 

### 3.3. Analysis of the Landscape Transition Matrix

The magnitude and the direction of changes in a landscape are the most important factors relating to landscape evolution. Aiming to analyze the change of landscape pattern in Jiulong River basin, we calculated the transition matrixes of five-time phases: 1990–1995 (Table 18), 1995–2000 (Table 19), 2000–2005 (Table 20), 2005–2010 (Table 21) and 2010–2015 (Table 22).

Source-sink landscape pattern changed violently during 1990–1995. Forestland, a sink landscape, transferred the largest area at 632.69 km^2^, of which 182.03 km^2^ of forestland were converted into a source landscape, residential land, while 182.43 km^2^ of forestland were converted to unused land, and other portions became cultivated land and water. The largest conversion rate belonged to the unused land, which is a sink landscape; the conversion area of unused land accounts for 30.78% of the total unused land area, followed by the source landscape, cultivated land. The area of the transferred-out cultivated land accounts for 27.63% of the total area of cultivated land.

270.43 km^2^ of forestland transformed into cultivated land and 518.02 km^2^ was converted into residential land during 1995–2000. The area of cultivated land transformed into forestland was 260.84 km^2^, and 154.92 km^2^ of cultivated land turned into residential land. 243.76 km^2^ of unused land turned into forestland, and 374.44 km^2^ turned into residential land. The transformed-out unused land area accounts for 59.98% of the total area of the unused land in Jiulong River basin. 

During the period from 2000 to 2005 in Jiulong River basin, as Table 20 showed, the landscape types with the largest areas of transition were forestland and cultivated land, including 461.52 km^2^ and 444.63 km^2^ respectively. 218.88 km^2^ of forestland was transformed into cultivated land and 210.42 km^2^ turned into residential land, while 218.71 km^2^ of cultivated land transferred to residential land, and 110.82 km^2^ of unused land transformed into residential land.

The area of transformed forestland was largest during 2005–2010 at 576.67 km^2^, and it mainly turned into cultivated land and residential land. The transition area of cultivated land, 588.01 km^2^, accounted for 32.32% of the area of cultivated land. In the process of landscape transition, the transformed area of residential land reached 478.97 km^2^, accounting for 19.31% of the total area of residential land, of which 252.69 km^2^ transferred into woodland. In the unused landscape, 112.15 km^2^ of the landscape turned into residential land, and the transition volume reached 69.13% of the total area of unused land.

The transition area of cultivated land was 588.01 km^2^, which mainly transferred to residential land (381.24 km^2^) and forestland (151.85 km^2^). The transition rate of cultivated land reached 41.88%. 478.97 km^2^ of residential land transferred to other types, including forestland and cultivated land. Only 44.01 km^2^ of unused land transformed into other landscape types, while the transition area accounted for 39.07% of the total area of unused land. Transition occurred among water, orchards and other landscapes, but the changed area is smaller in these categories.

Based on analysis of five transition matrixes from 1990–1995, 1995–2000, 2000–2005, 2005–2010 and 2010–2015, forestland occupied the largest area in Jiulong River basin and the transition area of forestland was largest among six landscape types. In particular, from 1995 to 2000, the area of forestland that transformed into residential land reached 518.2 km^2^. Forestland, one of the sink landscapes, mainly turned into source landscapes, including residential land and cultivated land, from 1990 to 2015, which was due to rapid development during the past three decades. The expansion of residential land led to the loss of cultivated land and unused land throughout the urbanization process during 1990–2015. Pressure to protect the forestland will increase continuously with further industrialization and development. 

The overall transition rate can be obtained on the basis of five transition matrixes.

(4) $$Transition\;rate=\frac{A_{In}}{Area}\;$$

Equation (4) gives the value of transition rate, where A_In_ is the sum of transform-in area of each landscape and Area is the total area of Jiulong River basin.

As Table 23 displayed, the overall transformation rate among landscape types in Jiulong River basin first increased and then decreased after 2000. The decreasing trend of the change rate tended to gradually stabilize. The highest transition rate appeared in 1995–2000, reaching 16.58%, which indicated that the most remarkable changes of the landscape occurred during this period. The transformation intensity decreased obviously after 2000, and the decreasing trend remained until 2015. 

### 3.4. Analysis of the Source-Sink Landscape Transition Matrix

We calculated the source-sink landscape transition matrixes of five-time phases: From 1990 to 1995 (Table 24), from 1995 to 2000 (Table 25), from 2000 to 2005 (Table 26), from 2005 to 2010 (Table 27) and from 2010 to 2015 (Table 28). The difference between area from sink landscape to source landscape and area from source landscape to sink landscape can reflect the change of source-sink landscape pattern of Jiulong River basin to some extent. The maximum difference appeared in 1995–2000, and the area transformed from sink landscape to source landscape was greatly larger than the area from source landscape to sink landscape in this period. 

## 4. Conclusions

The characteristics of the source-sink landscape pattern changes in the experimental basin were presented in this paper by using the classification of remotely sensed images coupled with GIS analyses. The random forest classifier, combined with textural features and spectral information, was proposed to improve the accuracy of classification in this paper. On the basis of classification maps from different periods, four landscape metrics were calculated to evaluate the landscape fragmentation. Five transition matrixes were obtained with the support of classified images to analyze the transition among different landscape types during the study periods. 

Jiulong River basin, located in southeast China, was selected as the study area in this paper. According to the landscape ecology and the standards of land-use classification, Jiulong River watershed was divided into six types: Forestland, water, orchards, cultivated land, residential land and unused land. Combined with the non-point source pollution process and source-sink theory, these six types were categorized into source landscapes (including cultivated land, orchards and residential land) and sink landscapes (including forestland, water and unused land), respectively. 

Forestland occupied more than 70% of the area of the whole watershed and was one of the dominant landscape types in this basin. The area of forestland decreased from 1990 to 2010 and began to increase after 2010. Residential land changed most rapidly during the study periods; its area in 2015 was two times larger than it was in 1990, which was a result of urbanization and industrialization. Water and orchards remained stable during the study period. The NP and PD of residential land and farmland increased from 1990 and began to decrease after 2005, which indicated an increasing degree of fragmentation of the two types during 1990–2005 and a decreasing extent of fragmentation from 2005 to 2015. The NP and PD of unused land decreased rapidly from 1995 to 2010, which was opposite to the trend of farmland and residential land. Sharp transitions appeared among the farmland, residential land and unused land and forestland. The transition area of forestland was consistently the largest. 

Results demonstrated that landscape metrics can allow landscape structure to be quantified and analyzed, and provide essential information on the spatiotemporal changes in landscapes. The transition matrixes reflect the transformation among different landscape types. The integrated evaluation of landscape change using landscape metrics and transition matrixes can characterize the evolution of source-sink landscapes. The integrated method can be used to analyze the evolution of source-sink landscape patterns in the watershed. The analysis results can provide support for allocation of the source-sink landscape pattern and land-use planning in the future, which is beneficial for the sustainable development of the watershed. 

Only four landscape metrics were calculated in this paper, but there are still a large number of metrics that can quantify the spatial characteristics of landscapes. Hence, further efforts should be made to apply additional landscape metrics for comprehensive analysis and select the appropriate indexes according to practical objectives. 

## Figures and Tables

**Table 1:** Sensors of the remote sensing images.

Image Acquisition Time	Sensors Information
1990	Landsat 5 TM
1995	Landsat 5 TM
2000	Landsat 5 TM
2005	Landsat 5 TM
2010	Landsat 5 TM
2015	Landsat 7 ETM+

**Table 2:** Description of each landscape type.

Landscape Type	Description
Forestland	A large area dominated by trees
Cultivated land	Land devoted to agriculture to produce food for humans
Residential land	Residential, commercial, industrial, transportation, mixed urban
Water	River, open water, lakes, ponds and reservoirs
Orchards	Land planted of trees or shrubs for food production
Unused land	Land areas of exposed soil and barren area

**Table 3:** Sequence number table of spectral and textural features.

Number		Mean	Variance	Homogeneity	Contrast	Dissimilarity	Entropy	Second Moment	Correlation
Band 1	1	7	8	9	10	11	12	13	14
Band 2	2	15	16	17	18	19	20	21	22
Band 3	3	23	24	25	26	27	28	29	30
Band 4	4	31	32	33	34	35	36	37	38
Band 5	5	39	40	41	42	43	44	45	46
Band 7	6	47	48	49	50	51	52	53	54

**Table 4:** Weights of the first 10 features in 1990.

Rank	Sequence Number	Weight	Rank	Sequence Number	Weight
1	4	0.0911	6	41	0.0671
2	33	0.0769	7	5	0.0670
3	49	0.0748	8	3	0.0621
4	17	0.0714	9	2	0.0583
5	6	0.0707	10	1	0.0575

**Table 5:** Weights of the first 10 features in 1995.

Rank	Sequence Number	Weight	Rank	Sequence Number	Weight
1	4	0.0856	6	1	0.0640
2	33	0.0798	7	6	0.0591
3	17	0.0786	8	5	0.0586
4	49	0.0673	9	25	0.0558
5	2	0.0655	10	9	0.0523

**Table 6:** Weights of the first 10 features in 2000.

Rank	Sequence Number	Weight	Rank	Sequence Number	Weight
1	33	0.0987	6	2	0.0613
2	4	0.0819	7	5	0.0578
3	6	0.0809	8	41	0.0563
4	49	0.0787	9	8	0.0518
5	1	0.0765	10	3	0.0511

**Table 7:** Weights of the first 10 features in 2005.

Rank	Sequence Number	Weight	Rank	Sequence Number	Weight
1	6	0.0859	6	4	0.0647
2	33	0.0780	7	2	0.0618
3	41	0.0744	8	25	0.0606
4	49	0.0724	9	9	0.0529
5	5	0.0667	10	1	0.0505

**Table 8:** Weights of the first 10 features in 2010.

Rank	Sequence Number	Weight	Rank	Sequence Number	Weight
1	41	0.1046	6	33	0.0597
2	49	0.0832	7	9	0.0500
3	5	0.0760	8	2	0.0447
4	17	0.0677	9	25	0.0410
5	6	0.0626	10	7	0.0396

**Table 9:** Weights of the first 10 features in 2015.

Rank	Sequence Number	Weight	Rank	Sequence Number	Weight
1	49	0.1040	6	6	0.0644
2	41	0.0994	7	17	0.0516
3	5	0.0872	8	4	0.0486
4	33	0.0686	9	25	0.0450
5	9	0.0656	10	1	0.0363

**Table 10:** Description of four landscape metrics.

Landscape Metrics	Description
Number of patches (NP)	The number of patches in the land-use type/landscape under investigation is counted.
Patch density (PD)	The number of patches per unit area in the landscape.
Mean patch size (MPS)	The average area of all patches in a landscape.
Percentage of landscape (PLAND)	The proportion of total area occupied by a particular land-use type.

**Table 11:** Assessment indexes of classification accuracy.

Year	1990	1995	2000	2005	2010	2015
Overall accuracy	86.32%	87.33%	87.64%	86.20%	89.45%	90.12%
Kappa coefficient	0.83	0.84	0.84	0.83	0.86	0.87

**Table 12:** The area of landscape change in the Jiulong River basin (km^2^).

	Year	1990	1995	2000	2005	2010	2015
Landscape							
Forestland		10,773.61	10,507.93	10,401.62	10,433.02	10,493.26	10,523.34
Cultivated land		1505.33	1359.67	1313.03	1345.12	1416.94	1016.12
Residential land		1081.04	1179.27	1838.68	2046.77	2094.79	2118.94
Water		283.10	414.53	383.42	282.34	287.45	368.19
Orchards		149.83	168.89	177.77	126.44	172.01	133.47
Unused land		772.33	935.55	450.09	330.90	112.75	407.28

**Table 13:** Number of patches (NP) for various types of landscape.

	Year	1990	1995	2000	2005	2010	2015
Landscape							
Forest land		23,586	28,053	33,355	22,536	22,149	20,640
Cultivated land		78,274	77,319	90,850	124,092	107,473	99,928
Residential land		72,400	65,866	79,901	96,736	84,623	74,384
Water		10,312	13,324	11,061	9186	12,932	21,080
Orchards		17,877	16,681	7920	15,191	19,517	18,490
Unused land		55,684	74,442	48,390	25,384	11,159	24,730

**Table 14:** Patch density (PD) for various types of landscape.

	Year	1990	1995	2000	2005	2010	2015
Landscape							
Forestland		1.61933	1.92594	2.29014	1.54731	1.51943	1.41687
Cultivated land		5.37403	5.30824	6.23772	8.52012	7.37268	6.85973
Residential land		4.97074	4.52195	5.48597	6.64186	5.80516	5.10622
Water		0.70799	0.91474	0.75944	0.63071	0.88714	1.44707
Orchards		1.22737	1.14521	0.54378	1.04301	1.33887	1.26928
Unused land		3.82307	5.11072	3.32244	1.74286	0.76551	1.69763

**Table 15:** Mean patch size (MPS) for various types of landscape (km^2^).

	Year	1990	1995	2000	2005	2010	2015
Landscape							
Forestland		0.45678	0.37457	0.31185	0.46295	0.47376	0.50985
Cultivated land		0.01923	0.01759	0.01445	0.01084	0.01318	0.01017
Residential land		0.01493	0.01790	0.02301	0.02116	0.02475	0.02849
Water		0.02745	0.03111	0.03466	0.03074	0.02223	0.01747
Orchards		0.00838	0.01012	0.02245	0.00832	0.00881	0.00722
Unused land		0.01387	0.01257	0.00930	0.01304	0.01010	0.01647

**Table 16:** Percent of landscape (PLAND) for various types of landscape.

	Year	1990	1995	2000	2005	2010	2015
Landscape							
Forestland		73.97%	72.14%	71.42%	71.63%	71.98%	72.24%
Cultivated land		10.34%	9.33%	9.02%	9.24%	9.72%	6.98%
Residential land		7.42%	8.10%	12.62%	14.05%	14.37%	14.55%
Water		1.94%	2.85%	2.63%	1.94%	1.97%	2.53%
Orchards		1.03%	1.16%	1.22%	0.87%	1.18%	0.92%
Unused land		5.30%	6.42%	3.09%	2.27%	0.77%	2.80%

**Table 17:** The annual water quality indices of two monitoring sites in Jiulong River.

Year	Monitoring Site	pH	DO (mg/L)	COD_Mn_ (mg/L)	TP (mg/L)	NH_3_-N (mg/L)
2005	Hua’an	6.727778	7.636857	2.136111	0.103694	0.249118
	Xiamen	7.217037	7.898888	1.833333	0.109118	0.315217
2006	Hua’an	6.851132	7.810577	2.973585	0.131019	0.375472
	Xiamen	7.289130	7.926739	2.047059	0.096077	0.266042
2007	Hua’an	6.733208	7.452453	2.277358	0.125942	0.316226
	Xiamen	7.093256	7.354186	2.600000	0.079107	0.373333
2008	Hua’an	6.75	7.19451	2.390196	0.114765	0.723333
	Xiamen	7.123111	7.346136	2.240476	0.120889	0.282857
2009	Hua’an	7.024894	6.884255	2.173077	0.105731	0.484419
	Xiamen	7.429608	7.310588	1.970000	0.075717	0.247843
2010	Hua’an	6.975577	7.745962	1.985385	0.111615	0.294019
	Xiamen	6.947115	7.676731	1.765490	0.054019	0.309077
2011	Hua’an	7.036078	6.095686	2.647647	0.105745	0.416078
	Xiamen	7.010208	7.113750	1.739521	0.063085	0.407292
2012	Hua’an	6.928	7.253333	2.416207	0.163133	0.438333
	Xiamen	7.233929	7.324138	2.165385	0.062690	0.387241
2013	Hua’an	7.009107	6.652321	1.680714	0.110232	0.646607
	Xiamen	6.802407	7.295000	2.510536	0.064714	0.417768
2014	Hua’an	6.730385	5.66	2.101923	0.111462	0.790192
	Xiamen	6.827115	7.724615	2.469231	0.073846	0.539346
2015	Hua’an	6.966731	6.853462	1.846154	0.101808	0.854615
	Xiamen	6.670588	6.723922	2.918039	0.082392	0.361647

**Table 18:** The landscape transition matrix from 1990 to 1995 (km^2^).

Year	1995							
	Landscape	Water	Forestland	Orchards	Cultivated Land	Residential Land	Unused Land	Total
1990	Water	227.01	33.93	0.12	6.04	10.46	5.54	283.1
	Forestland	155.76	10,140.92	10.42	102.05	182.03	182.43	10,773.61
	Orchards	0.01	8.81	132.34	4.62	0.12	3.92	149.82
	Cultivated land	3.47	225.89	22.52	1089.47	28.59	135.39	1505.33
	Residential land	15.17	54.21	2.47	32.95	900.01	77.11	1081.92
	Unused land	2.47	44.22	1.02	131.59	58.46	534.57	772.33
	Total	403.89	10,507.98	168.89	1366.72	1179.67	938.96	

**Table 19:** The landscape transition matrix from 1995 to 2000 (km^2^).

Year	2000							
	Landscape	Water	Forestland	Orchards	Cultivated Land	Residential Land	Unused Land	Total
1995	Water	244.77	135.63	0.04	0.72	22.42	0.86	404.44
	Forestland	103.21	9571.84	17.96	270.43	518.02	27.05	10,508.5
	Orchards	0.16	28.94	136.8	0.7	1.98	0.25	168.87
	Cultivated land	12.54	260.84	10.82	898.7	154.92	29.42	1367.24
	Residential land	12.46	154.61	2.44	68.61	923.04	18.07	1179.23
	Unused land	15.28	243.76	9.7	68.61	218.32	374.44	935.55
	Total	388.42	10,395.6	177.8	1313.2	1838.7	450.09	

**Table 20:** The landscape transition matrix from 2000 to 2005 (km^2^).

Year	2005							
	Landscape	Water	Forestland	Orchards	Cultivated Land	Residential Land	Unused land	Total
2000	Water	235.44	102.48	0.05	5.17	29.42	10.87	383.43
	Forestland	10.46	9943.07	4.37	218.88	210.42	17.39	10,404.6
	Orchards	0.25	36.82	106.87	14.28	17.46	2.08	177.76
	Cultivated land	1.77	193.17	3.84	868.85	218.71	27.14	1313.48
	Residential land	33.18	106.32	10.62	152.49	1459.7	72.55	1834.88
	Unused land	1.07	51.17	0.69	152.49	110.82	201.02	450.1
	Total	282.17	10,433	126.44	1345	2046.6	331.05	

**Table 21:** The landscape transition matrix from 2005 to 2010 (km^2^).

Year	2010							
	Landscape	Water	Forestland	Orchards	Cultivated Land	Residential Land	Unused Land	Total
2005	Water	196.24	45.18	0.42	5.46	34.4	0.63	282.33
	Forestland	33.88	9856.39	27.62	380.23	129.64	5.3	10,433.06
	Orchards	0.04	12.51	112.82	0.31	0.73	0.01	126.42
	Cultivated land	25.94	252.33	1.16	909.97	153.6	1.47	1344.47
	Residential land	25.58	252.69	29.73	85.09	1656.91	3.42	2053.42
	Unused land	5.77	74.03	0.35	35.88	112.15	101.89	330.07
	Total	287.45	10,493.1	172.1	1416.9	2087.43	112.72	

**Table 22:** The landscape transition matrix from 2010 to 2015 (km^2^).

Year	2015							
	Landscape	Water	Forestland	Orchards	Cultivated Land	Residential Land	Unused Land	Total
2010	Water	205.9	37.95	0.38	6.83	27.69	8.15	286.9
	Forestland	97.1	10,087.6	10.53	43.03	47.13	207.8	10,493.21
	Orchards	1.19	39.84	117.35	4.16	9.19	1.24	172.97
	Cultivated land	14.35	151.85	3.45	816.1	381.24	37.12	1404.11
	Residential land	48.97	202.87	2.13	140.66	1615.9	84.34	2094.88
	Unused land	1.02	2.78	0.14	2.72	37.35	68.64	112.65
	Total	368.53	10,522.9	133.98	1013.5	2118.5	407.3	

**Table 23:** Table of transfer rate of each time period in the overall landscape.

Period	1990–1995	1995–2000	2000–2005	2005–2010	2010–2015
Transition rate	10.58%	16.58%	12.01%	11.91%	11.35%

**Table 24:** The source-sink landscape transition matrix from 1990 to 1995 (km^2^).

Year	1995			
		Source	Sink	Total
1990	Source	2213.09	523.98	2737.07
	Sink	502.19	11,326.85	11,829.04
	Total	2715.28	11,850.83	

**Table 25:** The source-sink landscape transition matrix from 1995 to 2000 (km^2^).

Year	2000			
		Source	Sink	Total
1995	Source	2198.01	517.29	2715.3
	Sink	1126.22	10,716.84	11,843.06
	Total	3324.23	11,234.13	

**Table 26:** The source-sink landscape transition matrix from 2000 to 2005 (km^2^).

Year	2005			
		Source	Sink	Total
2000	Source	2852.82	473.28	3326.1
	Sink	732.31	10,572.97	11,305.28
	Total	3585.13	11,046.25	

**Table 27:** The source-sink landscape transition matrix from 2005 to 2010 (km^2^).

Year	2010			
		Source	Sink	Total
2005	Source	2950.32	573.99	3524.31
	Sink	726.15	10,319.31	11,045.46
	Total	3676.47	10,893.3	

**Table 28:** The source-sink landscape transition matrix from 2010 to 2015 (km^2^).

Year	2015			
		Source	Sink	Total
2010	Source	3090.18	581.77	3671.95
	Sink	175.8	10,716.94	10,892.74
	Total	3265.98	11,298.71	
